# Disability and disaster risk reduction as an incongruent matrix: Lessons from rural Zimbabwe

**DOI:** 10.4102/jamba.v11i1.648

**Published:** 2019-04-16

**Authors:** Wilfred Lunga, Paradzayi Pathias Bongo, Dewald van Niekerk, Charles Musarurwa

**Affiliations:** 1 Unit for Environmental Sciences and Management, African Centre for Disaster Studies, North-West University, Potchefstroom, South Africa; 2 Faculty of Social and Gender Transformative Sciences, Women’s University in Africa, Harare, Zimbabwe; 3 Department of Applied Education, Faculty of Education, Midlands State University, Gweru, Zimbabwe

## Abstract

The policy thrust of disaster risk reduction initiatives is in many instances tailored towards able-bodied people. This is at the expense of those challenged in many facets such as physically, mentally and other forms of disability. The suggestion for disability to be mainstreamed into disaster risk reduction initiatives has been made imperative by the late shift in hazard and disaster thinking at local, national and international levels. The increasing intensity and frequency of weather-related hazards and disasters, driven by climate change, has provided fertile ground on which mainstreaming ideas and policies have proved to be incongruent. This research paper focuses on two key topics in the disaster science field: pre-disaster risk reduction planning and post-disaster emergency response activities. It is based on experiences from disaster risk reduction projects in Bulilima and Mangwe districts in southern Zimbabwe between 2006 and 2014. A qualitative research methodology was employed, and data were collected using in-depth personal interviews, observations and focus group discussions. Both able-bodied people and people living with disabilities (PWDs) were part of the study. Main findings show that PWDs were not included in the formulation of disaster risk reduction measures. Other important findings were the variable nature of how people define or view disability, and even believe that disability has a purpose. Most of the so-called normal people lack the understanding of what constitutes a disaster to such an extent that even disability is regarded as a disaster. The paper unravels the need to have a relook that would bring PWDs into the centre of civil protection decision-making and policy formulation in Zimbabwe.

**Keywords:** disability; disaster risk reduction; disaster; vulnerability; Zimbabwe.

## Conceptualising disasters and disability

Hazards and disasters have immediate impact on a community, with regard to effects on resources and their influence on social life. They usually result in varying degrees of infrastructure and livelihood damage, human suffering, pain and even deaths (Roos, Chigeza & Van Niekerk 2010). The meaning of the terms ‘disaster’ and ‘hazard’ can sometimes be confusing because of their varied applications to describe disruptive phenomena. Nevertheless, understanding concepts is critical in appreciating the events and phenomena that negatively impinge upon our communities in various dimensions.

Disasters have been defined as potentially damaging physical events, phenomena or human activities that may cause fatalities or injuries, property damages, social and economic disruptions, or environmental degradation (ISDR 2010; Roos et al. 2010). Hazards can include latent conditions that may represent future threats and have different origins, whether natural (geological, hydrometeorological and biological) or induced by human activities (environmental degradation and technological hazards). Hazards can be single, sequential or combined (concatenated) regarding their origin and effects. Each hazard is characterised by its location, intensity, frequency and probability (Wisner et al. 2014a).

However, it is not the characteristics of hazards but exposure and vulnerability that determine the extent of a disaster. In a disaster, particularly poor people (Palakudiyil & Todd 2003) are hit hard as they tend to be the most vulnerable. Specific vulnerable groups include women, children and people with disabilities (Dunn, Uswatte & Elliott 2009; Guha-Sapir et al. 2012; Stough & Mayhorn, 2013). The more readily discerned disability forms include physical, sensory and mobility challenges, and the less obvious, perceptual, mental or cognitive impairments. According to Yeo (2001), disability is often considered as a specialist issue separate from mainstream Disaster Risk Reduction (DRR) issues as the history of disability in some cultures is little known. However, researchers such as Gaillard, Liamzon and Villanueva (2007), Lewis (1999) and Wisner, Gaillard and Kelman (2012) believe that it is not so much the vulnerability of people but rather the vulnerable conditions and exposure in which they find themselves, which add to disaster risk. While talking about a ‘natural disaster’ is common in English language, Benson, Twigg and Myers (2001) and Blaikie et al. (1994) argue that there is no such thing as a natural disaster, but there are natural hazards, such as cyclones, floods and earthquakes. The difference between a hazard and a disaster is an important factor. A disaster takes place when a community is affected by a hazard that overwhelms the capacity of the community to cope without external assistance. In other words, the impact of a disaster is determined by the extent of a community’s vulnerability to the hazard (exposure) (Wisner et al. 2014b) and the extent of its coping capacities. Vulnerability is not natural but a social state that is the result of a whole range of economic, social, cultural, institutional, political and psychological factors that shape people’s lives (Kelman & Stough 2015). In attempting to illustrate how a disaster risk is configured, Abarquez and Murshed (2004) point to the interplay of key disaster terms such as ‘vulnerability’, ‘resilience’, ‘capacity’ and ‘hazard’. Community vulnerability (Wisner et al. 2014b) exists when the elements at risk are in the path or area of the hazard and susceptible to damage caused by it. Similarly, Hemingway and Priestley (2014) noted that just as disability is not the inevitable outcome of functional impairment, human ‘disaster’ is not the inevitable outcome of natural ‘hazard’. Resilience plays a significant role in determining thresholds between ‘impairment’ and ‘disability’, and ‘hazards’ and ‘disasters’.

The United Nations International Strategy for Disaster Reduction (UNISDR 2015) defines resilience as the capacity of a system, community or society potentially exposed to hazards to adapt by resisting or changing in order to reach and maintain an acceptable level of functioning and structure. This supports what was noted earlier by Manyena, Fordham and Collins (2008), that resilience is determined by the degree to which a social system is capable of organising itself to increase its capacity to learn from past disasters for better protection in future and to improve risk reduction measures. In a similar vein, Dunn et al. (2009) and Stough & Mayhorn (2013) argued that people with disabilities show significant resilience tendencies in coping with their situations.

The definitions of disability vary according to regions, countries and contexts (Albert 2006). The United Nations Convention on the Rights of Persons with Disabilities (CRPD) states that persons with disabilities include those who have long-term physical, mental, intellectual or sensory impairments which in interaction with various barriers may hinder their full and effective participation in society on an equal basis with others (Voluntary Services Overseas [VSO] 2006:6).

‘Disability’ is subsumed by the broader category of ‘health’ (Dunn et al. 2009; Stough & Mayhorn, 2013). In the emergency management and disaster planning literature, ‘disability’ often appears in checklists and taxonomies of ‘social factors’ or ‘vulnerable groups’ that require special attention (Wisner et al. 2013). The essential elements include the long-term impairment of a physical, mental, intellectual and sensory (including hearing and/or speech impairment and visual impairment) nature.

Various organisations define disability in various ways. The *American Disabilities Act* passed in 1990 defines ‘disability’ as ‘a physical or mental impairment that substantially limits one or more of the major life activities’ (Charlton 1998). Voluntary Services Overseas explains disability as the disadvantage and exclusion which arise as an outcome of the interactions between people who have impairments and face social and environmental barriers because of the failure of society to take account of their rights and needs (VSO 2006:6). About 10% of the current global population (World Health Organization 2011) is classified as having some form of disability, with 80% found in developing countries. Interestingly, disability statistics in the United States exceeds 20% of the population (Brault 2012a, 2012b), indicating a better rate of disability identification rather than a greater incidence relative to other countries. In time, it may be expected that the World Health Organization estimate of the global population living with disability will grow depending on awareness and identification of disabilities in developing countries.

Disability has different meanings depending on the perspectives employed, that is, whether it is indigenous and non-indigenous. In some communities, disabilities have been regarded as gifts (Lovern & Locust 2013). Indigenous languages in some parts of the world do not have a word for the term ‘disability’ (Durst 2006), but the difference in the speed and course of developments is what makes an individual unique. Beliefs among vulnerable communities justify for the lack of indigenous vocabulary to describe disability. Scholars such as Coleridge (1993) discuss disability through models. On one hand, the ‘traditional model’, according to most religions and cultures, views disability as a form of punishment, hence the reason for social exclusion (Coleridge 1993, 2000). On the other hand, the ‘biomedical’, ‘medical’ or ‘individual’ model sees disability as a departure from ‘normal’ that needs to be ‘cured’; thus, it is more technocratic. The medical framework of disability speaks of an inability centred in the individual to support themselves or contribute to the society (Durst 2006). However, the ‘social’ model in Coleridge’s words, ‘starts from the point that integration is ultimately about removing barriers, not “normalization,” or cure, or care’. According to Coleridge (1993), it is politically vital to prove that disability is a socio-historical construct, an oppressive structure that was built and which therefore can be torn down and replaced with inclusive social relations. (p. 107)

Although mainstream development agencies still argue that they cannot include disability issues in their programmes because they are not disability ‘specialists’, disability issues are important in DRR, especially with regard to equality, empowerment, human rights, poverty and marginalisation (Brault 2012b). The other common model of disability views disability as what is ‘wrong’ with people who live with a disability and how their health is compromised (Hurst & Albert 2006). This view of disability is often called the medical model of disability, hence conceptualising disability as an individual health issue. It implies that persons living with disabilities become socially marginalised. Thus, people with disabilities may end up imagining themselves as damaged, abnormal, patients and/or dependent objects of a variety of medical or rehabilitative interventions. As the problem is primarily a medical one, the solution tends to be a cure and/or a rehabilitation.

Another model is the charitable model of disability which views people with disabilities as objects of pity who need the help of welfare approaches in times of disaster (Bankoff, Frerks & Hilhorst 2004; Reinhardt et al. 2011). There is no recognition of equal rights or the role that discrimination plays (Wisner et al. 2013). The implication is that people [*living*] with disabilities (PWDs) are seen as victims at the mercy of society’s charity. PWDs are viewed as suffering people to be pitied and cared for. Whatever is done for PWDs is done out of charity. The last model is the social model of disability. It defines the limitations imposed on people by social, cultural, economic and environmental barriers. Disability is not about health or pathology but about discrimination and social exclusion (Albert 2005). Albert (2006) perceives that removing barriers to exclusion and discrimination addresses issues of disability in planning for disasters.

The access of PWDs to services and participation is a right (Casey-Cannon, Nguyen & Velazquez 2005). Human rights, a 20th-century phenomenon, developed in response to the atrocities committed during the Second World War. They set out an internationally accepted moral code by which the intrinsic humanity of every individual is recognised and protected (Hurst & Albert 2006). As disability is being described as barriers faced by people with impairments to achieve equality and justice, and because PWDs are human beings too, it is needless to say that disability is a human rights issue; thus, ‘Nothing about us without us’ became a slogan promoted by Disabled Peoples’ International in 1981. The slogan has been particularly effective in capturing the key idea of the struggle for human rights – self-determination by PWDs.

How disability is defined has implications for practice and policy responses to the needs of persons living with disability (Yeo 2001; Yeo & Moore 2003). Levels of dealing with disabilities vary depending on whether ‘planning’ is linked to DRR and/or disaster response and recovery. It must be acknowledged that in Zimbabwe, emergency management does not take disability to be of a priority concern. Thus, this research revealed that benefits can be expected from full partnership with, and participation by, people with disabilities and their organisations in all phases of disaster risk management and planning.

## Dealing with disasters and disability

Natural and human-made disasters affect both the able-bodied and PWDs in the same way as witnessed in the aftermath of Hurricane Katrina in 2005, earthquake in Haiti in 2010 and the Nepal 2015 earthquake (Dahal 2016). These natural events resulted in phenomenal devastation in all areas within their path. From a disability perspective, disasters not only create disabilities but also inflict injuries to PWDs, thus adding more problems to the already disadvantaged individuals. This is compounded by the fact that the needs of people with disabilities are often not taken into account during disaster planning (Wisner 2012). People living with disabilities may require specific forms of evacuation and shelter after disruption of their social contacts and support network (Kumar et al. 2010). In South Africa, Dube (2007) examined key pieces of disability legislation and pointed out that while intentions have been excellent, delivery has been exceedingly poor. Consequently, policies have had little impact on the lives of people with disabilities. There are various capacity constraints at the programme level, poor championing and inadequate monitoring, as well as a lack of finance. According to Dube (2007), some countries’ progressive disability policies disappeared early in their existence. At the heart of the disability rights movement are legal challenges to the lack of ‘access’ and ‘equal opportunity’. Disaster risk reduction is constructed around the abilities and needs of people without impairments in mobility, hearing, sight, speech, stamina and cognition, mental or emotional stability. According to Dube (2007), Ingstad (1999) and Reinhardt et al. (2011), policies for DRR presume the dependence of the person with disabilities upon a caregiver and disregard situations in which there may not be anyone to assist the person in question, nor do they consider a person with disability as an asset. A case could be made that information on what to do in disaster situations ought to be directly available to persons with various types of impairments who may well have to ‘cope’ on their own. Advice could be provided about how a person with a disability could go about developing his or her own support system (Kailes 2005)^1^ and could be in a position to provide assistance and support to someone else (Fjord 2007; Kailes 2005).

Current disaster paradigms are biased towards helping the already privileged or physically abled. In short, PWDs are victims and are stigmatised as revealed by the findings of the United Nation (UN) Treaty on PWD. On 30 March 2007, the United Nations ratified the Treaty on the Human Rights of PWDs to protect and promote the rights of a million people living with disabilities. In using the term ‘vulnerability’ to conceptualise and assess, Wisner et al. (2007) propose a ‘move away from simple taxonomies or checklists of “vulnerable persons” to a concern with “vulnerable situations,” which people move into and out of over time’. By authorising this shift, the authors move in the direction that splits apart the embodied or social characteristics of an individual or group from social situations, causing differential burdens of harm because of barriers denying them access to social and material resources (Corker & Shakespeare 2002; Eide & Ingstad 2011; Ingstad & Whyte 2007).

## Research purpose and questions

This research is based on the researchers’ involvement and experiences in Disaster Risk Reduction (DRR) projects in Bulilima and Mangwe districts in southern Zimbabwe during 2006–2014. It was during their interaction with residents of the two districts that they realised that there were issues around the concepts – disability, DRR and inclusivity. Stemming from the interaction, the research set out to find out the extent to which PWDs were included in pre-DRR planning and post-disaster emergency response activities, as anecdotal evidence seemed to prove otherwise.

The guiding questions of this study were the following:

- What are the stakeholders’ understanding of the concepts – disability and disaster?
- How do policy provisions cater for PWDs in pre-disaster risk planning and response?
- To what extent are PWDs participating in community projects aimed at reducing the risk of disasters?

As these questions sought to solicit unquantifiable responses, opinions and experiences, a qualitative approach became the approach of choice.

### Methodology

An interpretivist–constructivist paradigm was used to guide the research; thus, qualitative data were collected to understand the phenomenon of non-inclusion of PWDs in DRR programmes. The interpretivist approach was suitable for this study as it regards reality as something that is not ‘out there’, as it exists in human mind and is conditional upon human experiences and interpretation (Lotz-Sisitka, Fien & Ketlhoilwe 2013). As reality is not independent but socially constructed and can have varied meanings, the rationale behind this qualitative research design was that it was the most suitable approach in understanding social or human problems, particularly those linked to DRR. Thus, appropriate data collection methods consistent with qualitative research such as individual interviews and focus group discussions were used to collect important information from participants, giving them an opportunity to express their views freely about the presence or non-presence of disability issues in DRR programmes since 2006 (Bogdan & Biklen 1997; González y González & Lincoln 2006; Lincoln & Guba 1985). Naturalistic Inquiry. Newbury Park, CA: Sage Publications.). Interviews were conducted in vernacular language, IsiNdebele, and recorded using digital voice recorders. This enabled the researchers to access participants’ feelings, intentions, perceptions, beliefs, knowledge and opinions. Informal interviews were also used along the course of the research. An effort was made to interview most community stakeholders such as councillors, chiefs and village heads at district level, chief executive officers, staff of the two rural district councils (RDCs), district administrators (DAs), extension workers and members of non-governmental organisations (NGOs). Interview data were analysed for experiences, opinions and attitudes of participants in relation to DRR programmes that were implemented by RDCs in partnership with NGOs in Bulilima and Mangwe where the research was carried out.

Three focus groups with an average of 21 participants each, comprising ward leadership, men, women, youths and the elderly, were also engaged with the purpose of establishing how far people with disabilities were being integrated into DRR programmes. People with disabilities were included in the focus groups, and this gave them an opportunity to discuss freely and objectively about their inclusion or non-inclusion in disaster risk–related matters. However, it is important to note that many of those with disabilities that were included in the focus groups were of a physical nature, and mostly such disabilities were a direct result of their engagements in the liberation war. Very few had congenital disabilities. Participants gave insights on the variance of opinions ranging from the empirical reality to policy and institutional issues and challenges in the two districts. The use of focus group discussions and individual interviews, as well as a wide range of participants or stakeholders, enabled the researchers to triangulate the data and hence ensuring the trustworthiness of the findings.

The study area, Bulilima and Mangwe in Matabeleland South Province, Zimbabwe, is characterised by recurring disasters associated with droughts, animal disease–associated insect infestation and high levels of food insecurity (Government of Zimbabwe and United Nations Development Programme 2012). This is against a background where it is regarded as one of the driest regions in the country, making cattle ranching as the main livelihood. Rain-fed agriculture is not profitable and is often not an option in the area. The situation is further compounded by the fact that there are few water reservoirs as it is a low-rainfall area. Thus, some households have diversified out of agriculture into harvesting forest products for both subsistence and commercial purposes. Like any other province of Zimbabwe, disasters in Matabeleland South also have been compounded by HIV and AIDS pandemic, thereby lowering productivity.

### Summary of findings

One important finding of this study was that disability issues were not reflected in DRR programmes in Zimbabwe. Policy documents on DDR and implementation documents from RDCs and NGOs are silent about how people living with various disabilities should be handled whenever a disaster such as a drought or flood occurs. This was consolidated by information from focus group discussions with individuals with disabilities intimated that they were never consulted and involved in DRR projects from planning to implementation. A good example is that of a livelihood-centred project undertaken in one of the districts, where stakeholders were supposed to buy-in after awareness raising was implemented. This is despite the claim by officials in other focus groups that there were extensive field consultations and verifications among stakeholders in the two districts prior to implementation.

Another finding is the variable nature of how people define or view disability, and even believe that disability has a purpose. One elderly person with a disability stated that disabilities are gifts from the Creator, sent to the community for a specific reason. The elderly participant went on to state that PWDs have a purpose, value and that they belong to the community and should be involved in every community programme. Research participants were also of the perception that PWDs have unique abilities that each of them brings to the world; and have something to offer thus they should be honoured for who they are and how they can contribute to the larger society. However, within the dominant DRR system, they felt that their unique abilities are not always valued or even recognised.

The third and a very important finding is, and interestingly, that most of the so-called normal people lack the understanding of what constitutes a disaster to such an extent that even disability is regarded as a disaster, and hence it was thought that disability should be planned for just like any other disaster. Development agents’ personnel and district officials working with communities in the study area believe that disability is synonymous with inability to do something. However, although district officials and traditional leaders revealed that DRR awareness workshops were planned for and held consistently, the interests of PWDs and the people themselves were often not included. Even for some development projects such as strengthening of soils, water conservation, exploring livelihood strategies away from agriculture, hazard awareness education and DRR strategies to combat droughts and floods, people with disabilities were not involved. In all cases, it proved that projects were for and by the able-bodied people; hence, one female with a disability had this to say:

I do not accept the notion that am not able to always fully participate in projects that help us develop the community. I as an individual can contribute something to be learned as a member of the community. (Participant 1, female, 53 years, grade 7 [primary school] [authors own translation])

The implication of these findings clearly shows that disaster reduction programmes were not inclusive of PWDs and thus did not effectively promote both participation and socio-economic independence of PWDs. District councils through the Ministry of local government have always been involved in initiatives such as ‘drought relief’, ‘food for work’ and ‘food for assets’. Although the central theme of these initiatives was their emphasis on Community-based Disaster Risk Management (CBDRM), CBDRM has been defined as a process of disaster risk management in which at-risk communities are actively engaged in the identification, analysis, treatment, monitoring and evaluation of disaster risks to reduce their vulnerabilities and enhance their capacities (Shaw 2012). Conclusions from the field reveal that other members of the community were excluded. One elderly woman with disability had this to say:

During the time of bad harvests the Department of Social Services provides grain to all affected villages. However, in most cases people are required to go and get the food from a central point and yet nothing is done to bring the food to the persons living with disabilities. (Participant 2, female, 63 years, grade 4 [primary school] [authors own translation])

This implies the people at the heart of decision-making and implementation of disaster risk management activities, and obviously those with disabilities, are not working together (Reinhardt et al. 2011). This defies the logic of Participatory Disaster Risk Assessment (PDRA), which is a major stage after the consultative phase like the one identified earlier. This reveals that PWDs are left out right from the planning stage and hence this affects the programme implementation as well. Disability is generally regarded as a flaw of the individual, thus presenting a barrier to effective development, equity, and inclusion. However, it could be that disabilities vary in nature and magnitude and thus it may lead to oversights on the part of officials. Another dimension could be that there is a lack of a database for disabled people which chronicles the nature of disability, where they are located and specify their needs. From a DRR point of view, there could be a requirement for a dedicated office that can cater for the needs of PWDs.

### Integrating disability into disaster risk reduction: Reality or illusion

While this research has been referring to the Bulilima and Mangwe as communities, this is obviously a myopic view of what a community is. While the concept of community has always been associated with a geographical entity such as a country, village or city, communities can also be virtual communities that exist as mental spaces and can also be formal or informal (Musarurwa 2012). People with disabilities are a good example. Although they are an entity with their own interests, their numbers do not make their physical location viable to be considered as a separate entity, and hence they are excluded from most social, developmental and DRR programmes. Disability cuts across intellect, age, sex, race and religion, and hence this diversity of facets makes it nearly impossible to talk of meaningful inclusion. While people with disabilities have rights like any other individuals, and communities preach about equality in every sense, it is evident from this discussion that including disability issues in DRR in Zimbabwe is more of an illusion than a reality. It will require great effort and time to adequately address disability issues.

The ideation and implementation of DRR projects in southern Zimbabwe raises a number of issues in light of disability. People with disabilities in communities feel dismissed by government officials and by their own community or do not feel well-equipped to talk about disaster issues that they experience. Notwithstanding the fact that the DRR initiatives discussed earlier were premised on the need to target vulnerable communities, its interventions were glaringly short of addressing priorities and needs of PWDs. This raises real concerns about the inclusiveness of DRR in communities where the study took place. Faced with recurring climatic disasters costing the lives and livelihoods of people, especially vulnerable people like those with disabilities, this should be a cause for concern.

However, mainstreaming disability would have yielded more positive results from a human rights perspective, which requires equal treatment, all socio-economic groups and participation of every individual. Other findings common across all the groups that took part were that vulnerability capacity assessments conducted were characterised by the absence of people with disabilities. Based on the generally accepted assumption that at least one in 10 people have some form of disability, a tenth of the population is still too large a number to be ignored (Zola 2005). There is a greater possibility that most PWDs could not attend planning workshops mainly because of mobility issues, or that mobilisation to attend workshops was not inclusive. Discussions with research participants revealed that even in few instances where disability issues were raised, facilitators indicated that they were not capacitated enough and trained to handle disability issues. The parties within communities are failing to take all necessary measures to ensure the protection and safety of people with disabilities during disasters. The study revealed that the effective and meaningful participation of people with disabilities in all DRR initiatives ensuring empowerment has not been the key solution among implementation stakeholders in Bulilima and Mangwe as defined in the Sendai Framework Section V 36(a)(iii). Finally, the study confirms what has been seen in the literature that people identified as having a disability still face barriers in accessibility to DRR programmes.

## Conclusion

In brief, it is apparent from the discussions that took place in Bulilima and Mangwe that the root cause of inclusion and/or exclusion lies in the paradigms that the various groups use to define disability. On one hand, people view disability as a sickness or condition that needs a cure. Therefore, PWDs need prescriptions to improve their lives, and as such there is no need to consult and include them in DRR, in as much as patients cannot decide how a doctor can attend to illness. This is a philosophical short sight. On the other hand, those with disabilities seem to confirm that disability is a social construction. They can do something meaningful like taking part in DRR activities. Thus, they need to be included in disaster risk initiatives as they are the most vulnerable group besides women, children and the elderly subgroups. This is worsened by major institutional drivers of DRR interventions at provincial, district and ward levels that are all composed of people without disabilities. It, therefore, follows that DRR policies and strategies adopted by these institutions were devoid of how to mainstream disability.

Based on these findings, one of the recommendations of this study is that there should be a political mechanism to remove obstacles to inclusionary planning. Although ‘empowerment’ is regarded as a key concept in the social movements of minorities (Coleridge 1993), people with disabilities would have been taken seriously only if they constituted a significant political constituency. There is a need for line agencies working in DRR issues to modify their programmes targeted for vulnerable communities; hence this study advocates for a paradigm that brings people with disabilities at the centre of DRR decision-making and policy formulation in Zimbabwe.

Another recommendation is the need for support that builds and sustains the capacity of people living with disabilities and not the capacity of larger development agencies acting as intermediaries. Governments and development agencies should tackle the challenges of policy formulation, which inhibit the implementation of policies on mainstreaming disability in DRR. It is hoped that this research will help facilitate disability inclusion in policy-making processes. To achieve equality, therefore, means removing the social, cultural and environmental conceptualisation of disability from the human rights approach, which is a strategy for dealing with discrimination and social exclusion faced by people with disabilities (Miller & Albert 2005). Areas for further research are to investigate institutional development support for disaster risk management authorities on frameworks that can be used to approach most vulnerable communities that include PWDs. Many vulnerable groups tend to be closed and fail to be selected in DRR and development programmes.

## Footnotes

1 Kailes provides excellent ‘Earthquake Tips for People with Disabilities’, for example, on establishing a personal support network, preparing lists of emergency health needs and emergency contacts, conducting an ability self-assessment, ensuring maintenance of 7–14 days’ medication supply, keeping important equipment and assistance devices in secure places for quick and easy location, etc. There are also earthquake tips for people with differing specific disabilities.
